# Mouse genetics identifies unique and overlapping functions of fibroblast growth factor receptors in keratinocytes

**DOI:** 10.1111/jcmm.14871

**Published:** 2019-12-12

**Authors:** Michael Meyer, Maya Ben‐Yehuda Greenwald, Theresa Rauschendorfer, Catharina Sänger, Marko Jukic, Haruka Iizuka, Fumimasa Kubo, Lin Chen, David M. Ornitz, Sabine Werner

**Affiliations:** ^1^ Department of Biology Institute of Molecular Health Sciences ETH Zurich Zurich Switzerland; ^2^ Center of Bone Metabolism and Repair Department of Rehabilitation Medicine State Key Laboratory of Trauma, Burns and Combined Injury Trauma Center Research Institute of Surgery Daping Hospital Third Military Medical University Chongqing China; ^3^ Department of Developmental Biology Washington University School of Medicine St. Louis Missouri

**Keywords:** atopic dermatitis, epidermal barrier, epidermis, FGF, FGFR, fibrosis, skin

## Abstract

Fibroblast growth factors (FGFs) are key regulators of tissue development, homeostasis and repair, and abnormal FGF signalling is associated with various human diseases. In human and murine epidermis, FGF receptor 3 (FGFR3) activation causes benign skin tumours, but the consequences of FGFR3 deficiency in this tissue have not been determined. Here, we show that FGFR3 in keratinocytes is dispensable for mouse skin development, homeostasis and wound repair. However, the defect in the epidermal barrier and the resulting inflammatory skin disease that develops in mice lacking FGFR1 and FGFR2 in keratinocytes were further aggravated upon additional loss of FGFR3. This caused fibroblast activation and fibrosis in the FGFR1/FGFR2 double‐knockout mice and even more in mice lacking all three FGFRs, revealing functional redundancy of FGFR3 with FGFR1 and FGFR2 for maintaining the epidermal barrier. Taken together, our study demonstrates that FGFR1, FGFR2 and FGFR3 act together to maintain epidermal integrity and cutaneous homeostasis, with FGFR2 being the dominant receptor.

## 1 INTRODUCTION

Fibroblast growth factors (FGFs) are master regulators of development and tissue repair, and abnormal expression of FGFs or their receptors is associated with a wide variety of human diseases.^1^, ^2^, ^3^ Most of the 22 members of the mammalian FGF family bind and activate four receptor tyrosine kinases, designated FGFR1‐FGFR4,^3^ which exert distinct functions in different tissues and organs. In some cases, however, partially overlapping functions of FGFRS have been discovered as revealed by a stronger phenotype of double‐ compared with single‐knockout mice. This was previously demonstrated in our laboratory for the skin through generation and characterization of mice lacking FGFR1 or FGFR2 or both receptors in keratinocytes.^4^ Whereas mice lacking only FGFR1 in this cell type are phenotypically normal, mice lacking FGFR2 in keratinocytes develop hair follicle and sebaceous gland abnormalities and mild, although progressive skin inflammation.^4^, ^5^ Interestingly, loss of both receptors resulted in a strong phenotype characterized by progressive loss of hair follicles and sebaceous glands and development of chronic skin inflammation.^4^, ^6^, ^7^ This finding demonstrates that FGFR1 signalling is unmasked in the absence of FGFR2, probably through FGF10/FGF22 signalling to the b splice variant of FGFR1.^2^, ^4^ The phenotype of the double mutant mice (designated K5‐R1/R2 mice) shows many similarities to the skin inflammation that occurs in patients with the chronic inflammatory skin disease atopic dermatitis (AD).^4^, ^6^, ^7^, ^8^ It results from a defect in the epidermal barrier that is caused at least in part by decreased expression of major tight junction components that are under direct control of FGFR signalling in keratinocytes,^4^, ^6^, ^7^, ^8^ We showed that different types of immune cells are attracted and/or activated upon establishment of the barrier defect, and growth factors and cytokines secreted by immune cells and fibroblasts caused keratinocyte hyperproliferation and epidermal thickening.^4^, ^6^, ^7^, ^8^ Nevertheless, the double‐knockout mice have a normal life expectancy and the inflammatory phenotype remains surprisingly mild under non‐challenged conditions. This suggested that other FGF receptors or receptors for different growth factors can compensate at least in part for the loss of FGFR1 and FGFR2 in keratinocytes. As FGFR3, in particular the FGFR3b splice variant, is also expressed in the murine epidermis,^9^ we speculated that loss of this receptor in K5‐R1/R2 mice would aggravate the phenotype. In addition, a phenotype in mice lacking only FGFR3 in keratinocytes was anticipated, as activating mutations in the *FGFR3* gene are the cause of the genetic skin disorder acanthosis nigricans and also induce seborrhoeic keratosis and epidermal naevi.^10^, ^11^, ^12^, ^13^ Here, we show, however, that loss of FGFR3 in keratinocytes does not obviously affect skin morphogenesis, homeostasis or wound repair in mice. Surprisingly, loss of all FGF receptors in keratinocytes is compatible with life, but the FGFR3 deficiency further aggravated some of the phenotypic abnormalities seen in K5‐R1/R2 mice. Overall, these results identify FGFR2 as the major functional FGF receptor in keratinocytes, whereas FGFR1 and FGFR3 have a “back‐up” function.

## 2 MATERIALS AND METHODS

### 2.1 Mice

**Figure 1:**
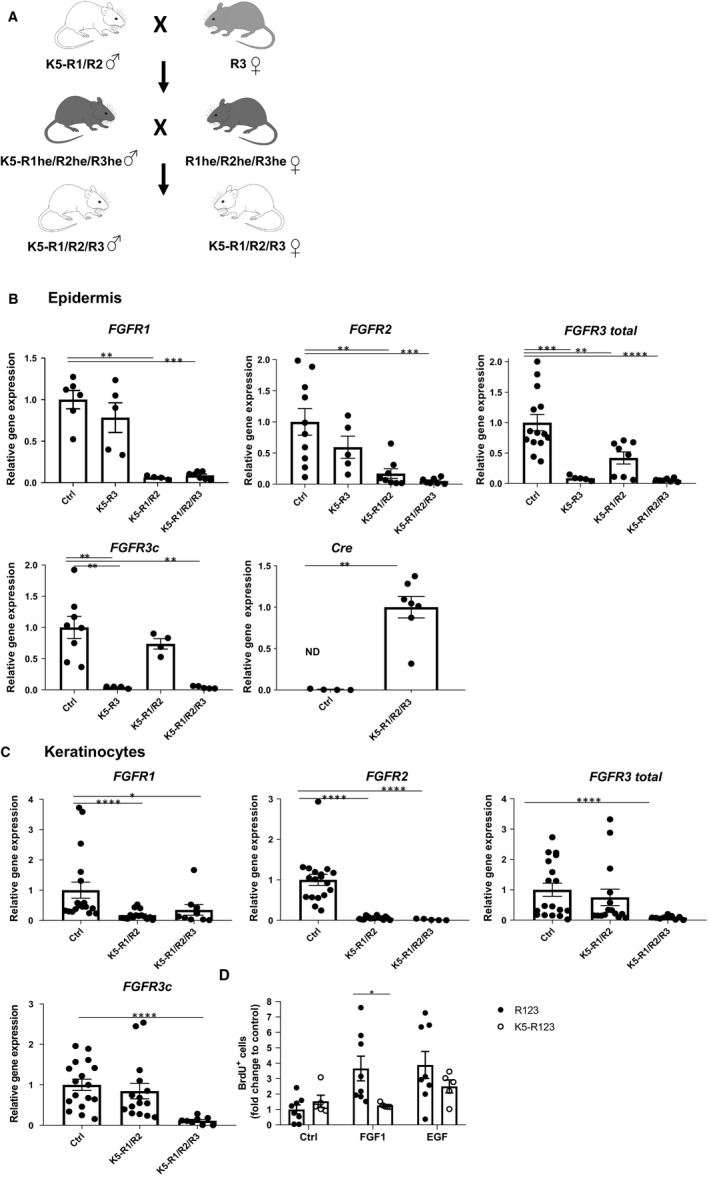
Verification of the *FGFR3* knockout in the mutant epidermis and in isolated primary keratinocytes A, Breeding scheme for the generation of mice lacking FGFR1, FGFR2 and FGFR3 in keratinocytes (K5‐R1/R2/R3 mice). He = heterozygous. B, qRT‐PCR analysis of RNA samples from isolated epidermis of adult Ctrl, K5‐R3, K5‐R1/R2 and K5‐R1/R2/R3 mice for *FGFR1*, *FGFR2*, *FGFR3* (all isoforms), *FGFR3c* and *Cre* relative to *Rps29* as indicated. C, qRT‐PCR analysis of RNA samples from primary keratinocytes derived from 3‐day‐old Ctrl, K5‐R1/R2 and K5‐R1/R2/R3 mice for *FGFR1*, *FGFR2*, *FGFR3* (all isoforms) and *FGFR3c* relative to *Rps29* as indicated. D, Primary keratinocytes from K5‐R1/R2/R3 or Ctrl mice were incubated overnight in keratinocyte serum‐free medium without EGF and subsequently treated with 10 ng/mL FGF1 or EGF for 24 hours and analysed for BrdU incorporation. Bars indicate mean ± SE. The mean value of the Ctrl mice was set to 1. N = 4‐11 per genotype. *P ≤ .05, **P ≤ .01, ***P ≤ .001, ****P ≤ .00 = 1. Mann–Whitney *U* test

Mice lacking FGFR1 and FGFR2 in keratinocytes (K5‐R1/R2 mice) were previously described.^4^, ^6^, ^7^, ^8^, ^14^ To generate mice lacking a functional FGFR3 protein in keratinocytes, we mated mice with floxed *FGFR3* alleles^15^ with K5‐Cre mice.^16^ Triple mutant mice were obtained by crossing females with floxed *FGFR3* alleles with K5‐R1/R2 males (Figure 1A). The F1 generation was paired *inter se* until K5‐R1/R2/R3 mice were obtained as described in Figure 1A. All K5‐Cre mice used for breeding were males, as global deletion occurred with females.^16^ Because of the difficult breeding scheme, each experiment included mice from different litters, but at least 1‐2 mice from the same litter were used for a direct comparison in all experiments. All mice were in C57BL/6 genetic background. Control mice (Ctrl) were mice with floxed *FGFR* alleles but without Cre recombinase or occasionally K5‐Cre mice. They were housed under specific pathogen‐free conditions and received food and water ad libitum. Mouse maintenance and all animal experiments had been approved by the veterinary authorities of Zurich, Switzerland (Kantonales Veterinäramt Zürich).

### 2.2 Establishment and culture of primary mouse keratinocytes

Keratinocytes were isolated from single mice as described previously^4^ and cultured in defined keratinocyte serum‐free medium (Invitrogen) supplemented with 10 ng/mL epidermal growth factor (EGF), 10^−10^ mol/L cholera toxin and 100 U/mL penicillin/100 μg/mL streptomycin (all from Sigma) in keratinocyte medium.^17^ Plates were coated with collagen IV (2.5 g/cm^2^) prior to seeding of the cells.

### 2.3 5‐Bromo‐2′‐deoxyuridine (BrdU) incorporation assay

Primary keratinocytes were incubated overnight in keratinocyte serum‐free medium without EGF. EGF (Sigma) or FGF1 (Peprotech) was added to a final concentration of 10 ng/mL and incubated for 24 hours. After 20 hours, BrdU (Sigma) was added to the cell culture medium at a final concentration of 100 μmol/L followed by incubation for 4 hours at 37°C and 5% CO_2_. Then, cells were washed with PBS and fixed with 4% paraformaldehyde for 30 minutes at RT. Afterwards, they were permeabilized and DNA was denatured using 0.1% Triton X‐100 in 2 mol/L HCl for 30 minutes. Cells were then incubated in boric buffer (100 mmol/L boric acid, 75 mmol/L NaCl, 25 mmol/L sodium tetraborate, pH 8.5) for neutralization for 5 minutes and blocked using 1% BSA for 30 minutes, followed by incubation with a FITC‐coupled anti‐BrdU antibody (11202693001, Sigma) at 4°C overnight. Cells were then washed with PBS, and a Cy2‐conjugated secondary antibody (1:500, Jackson ImmunoResearch Laboratories, Inc) was added to amplify the signal. Nuclei were counterstained with Hoechst 33342 (1:3000, Sigma).

### 2.4 Histology

Skin samples were fixed overnight with 4% paraformaldehyde (PFA) or 95% ethanol/1% acetic acid prior to paraffin embedding. Sections (7 μm) were stained with haematoxylin and eosin (H&E), or using the Herovici procedure.^18^ Visualization of mast cells was carried out using toluidine blue staining.^4^ Stained sections were analysed with an Axioskop 2 microscope equipped with a Plan‐Neofluar objective (20×/0.5NA) and photographed with an Axiocam HRc camera (all from Carl Zeiss, Inc). The Axiovision 4.6 software (Carl Zeiss, Inc) was used for acquisition of data. Data were analysed using ImageJ software. All analyses were performed blinded with regard to the genotype of the mice.

### 2.5 Immunohistochemistry and immunofluorescence analysis

Paraffin sections were dewaxed and rehydrated using a xylene/ethanol gradient followed by antigen retrieval using citrate buffer (10 mmol/L citric acid, pH 6.0) at 95°C for 1 hour and three washes with PBST (PBS, 0.1% Tween). Skin sections were then blocked with PBS containing 12% BSA for 1 hour at RT, followed by incubation with the primary antibodies overnight at 4°C. Cryosections were fixed in 1%‐4% paraformaldehyde for 10 minutes at RT, washed for 2 × 5 minutes in PBS and blocked in 5% BSA for 1 hour at room temperature, followed by incubation with the primary antibodies overnight at 4°C. For bright‐field microscopy analysis, a biotin‐conjugated secondary antibody, the Vectastain ABC Kit and the DAB peroxidase substrate kit (both from Vector Laboratories) were used for visualization. For immunofluorescence staining, slides were incubated at room temperature for 1 hour with the Cy2‐ or Cy3‐conjugated secondary antibodies (Jackson ImmunoResearch Laboratories, Inc) and counterstained with Hoechst 33342 (Sigma). The following antibodies were used for immunohistochemistry or immunofluorescence:

**Table:** 

Antibody	Source	Identifier
Rabbit anti‐Ki67	Abcam, Cambridge, UK	Cat#Ab15580; RRID:AB_443209
Biotinylated anti‐rabbit IgG	Jackson ImmunoResearch	Cat#111‐065‐003; RRID:AB_2337959
Rabbit anti‐keratin 6	BioLegend, San Diego, CA	Cat#PRB‐169P‐100; RRID:AB_10063923
Mouse anti‐keratin 10	DAKO, Glostrup, Denmark	Cat#M7002
Rabbit anti‐keratin 14	BAbCo, Richmond, CA	Cat#PRB‐155P; RRID:AB_292096
Rabbit anti‐loricrin	BioLegend	Cat#PRB‐145P; RRID:AB_10064155
Rabbit anti‐vimentin	Abcam	Cat#ab92547; RRID:AB_10562134
Anti‐rabbit Cy3	Jackson ImmunoResearch	Cat#711‐165‐152; RRID:AB_2307443
Anti‐mouse Cy3	Jackson ImmunoResearch	Cat#715‐165‐150; RRID:AB_2340813
Anti‐rabbit Cy2	Jackson ImmunoResearch	Cat#111‐225‐003; RRID:AB_2307385

### 2.6 Analysis of transepidermal water loss (TEWL)

Mouse back skin was shaved one day before TEWL analysis. TEWL was determined using a Tewameter (Courage and Khazaka Electronic GmbH) as described previously.^6^ Twenty‐five consecutive measurements were taken from four different places on the back on different days.

### 2.7 Isolation of RNA from mouse skin

Mice were killed, shaved, and epidermis from mouse back skin was separated from the dermis after heat shock treatment (30 seconds at 60°C in PBS followed by 1 minute at 4°C in PBS). Isolated epidermis and dermis were immediately frozen in liquid nitrogen. For RNA isolation, we used TRIzol® according to the manufacturer's instructions (Cat# 15596, Life Technologies).

### 2.8 Real‐time RT‐PCR

RNA was reverse‐transcribed using the iScript™ cDNA Synthesis Kit (Cat# 1708890, Bio‐Rad). Quantitative real‐time RT‐PCR (qRT‐PCR) was performed as described in the manual of the Light Cycler 480 II (Roche). The reverse transcription product obtained from 25 ng RNA was used together with 5.5 μL of SYBR Green Reaction Mix including 0.45 μmol/L forward and reverse primer mix. The reaction was performed in 50 cycles (95°C for 10 minutes for initial denaturation; 95°C for 10 seconds, 60°C for 20 seconds and 72°C for 20 seconds for each cycle). Samples were analysed in duplicates. Data were quantified using second derivative maximum analysis and gene expression represented as relative to the mRNA encoding ribosomal protein S29 (Rps29).

The following primers were used for qRT‐PCR:

**Table:** 

Target gene	Forward sequence 5′→3′	Reverse sequence 5′→3′
*Cldn1*	CTTCTCTGGGATGGATCG	GGGTTGCCTGCAAAGTACTGT
*Cldn3*	GCGGCTCTGCTCACCTTAGT	GACGTAGTCCTTGCGGTCGTA
*Cldn8*	TCAGAATGCAGTGCAAGGTC	AGCCGGTGATGAAGAAGATG
*FGFR1*	CAACCGTGTGACCAAAGTGG	TCCGACAGGTCCTTCTCCG
*FGFR2*	ATCCCCCTGCGGAGACA	GAGGACAGACGCGTTGTTATCC
*FGFR3 total (exons 2/3)*	GTG GCT GGA GCT ACT TCC GA	ATC CTT AGC CCA GAC CGT GG
*FGFR3c*	ACT CAA GAC TGC AGG CGC TA	GTC CTC AAA GGT GAC ATT GTG C
*FGFR4*	TTG GCC CTG TTG AGC ATC T	GCC CTC TTT GTA CCA GTG ACG
*Fst*	AGGGAAAGTGTATCACAAAGT	GAGTTGCAAGATCCAGAATG
*Il36b*	GCCTGTCATTCTTAGCTTGAT	TGTCTACTTCCTTAAGCTGC
*Inhba*	GGA GAA CGG GTA TGT GGA GA	ACA GGT CAC TGC CTT CCT TG
*Ocln*	TTG AAG TCC ACC TCC TTA CAG A	CCG GAT AAA AAG AGT ACG CTG G
*Rps29*	GGTCACCAGCAGCTCTACTG	GTCCAACTTAATGAAGCCTATGTCC
*S100a8*	GCCGTCTGAACTGGAGAAG	GTGAGATGCCACACCCACTTT
*S100a9*	CGCAGCATAACCACCATCAT	AAGATCAACTTTGCCATCAGC
*Tgfb1*	AGC CCG AAG CGG ACT ACT AT	TCC ACA TGT TGC TCC ACA CT
*Tgfb3*	CTC TGG GTT CAG GGT GTT GT	AAC CTG GAG GAG AAC TGC TG
*Cre*	AAC ATG CTT CAT CGT CGG	TTC GGA TCA TCA GCT ACA CC

### 2.9 Isolation of skin cells for flow cytometry

Mice were killed, shaved, and the back skin was harvested. The subcutaneous fat was scraped off. Epidermis and dermis were separated using 0.25% dispase (Gibco) for 50 minutes at 37°C under continuous shaking. The epidermis was treated with 100 Kunitz U/mL DNase (Sigma) in RPMI supplemented with P/S, 10% FBS and 20 mmol/L HEPES for 45 minutes at 37°C under continuous shaking. The dermis was cut into small pieces and treated with 0.25 mg/mL TL Liberase in RPMI supplemented with P/S and 20 mmol/L HEPES for 1 hour at 37°C under continuous shaking. Single cell suspensions were prepared by passing mixtures through 70‐μm cell strainers, and cells were washed 3x with 1x PBS.

### 2.10 Flow cytometry

Unspecific binding sites were blocked using a CD16/CD32 antibody. Dead cells were stained with Zombie Aqua™ dye (BioLegend). The antibodies used for flow cytometry analysis are listed below. Stained cells were quantified using a BD Fortessa machine and BD FACSDiva^TM^ 6.0 software (both from BD Biosciences). FlowJo v10 software (FlowJo) was used for data analysis. Compensation was performed with single‐colour controls. Compensation matrices were calculated using FlowJo v10 software and applied. Doublets and dead cells were excluded from the analysis. Fluorescence minus one (FMO) controls were used for gating analyses to distinguish positively from negatively stained cell populations.

The following dyes and antibodies were used for flow cytometry analysis:

**Table:** 

Antigen	Clone	Fluorophore	Dilution	Source
CD45	30‐F11	PB	1:400	BioLegend
Fc block (CD 16/32)	2.4G2	NA	1:100	BD BioSciences
CD3	17A2	BV785	1:300	BioLegend
I‐A/I‐E	M5/114.15.2	PE	1:2000	BD Biosciences
TCRδ	GL3	FITC	1:400	BD Biosciences

### 2.11 Wound healing experiments

Female K5‐R3 and Ctrl mice (9‐11 weeks of age) were anaesthetized by intraperitoneal injection of ketamine/xylazine (100 mg ketamine/5‐10 mg xylazine per kg bodyweight). After shaving of the back skin, four full‐thickness excisional wounds of 5 mm diameter were generated using a biopsy punch, two wounds on each side of the dorsal midline. Wounds were allowed to heal without dressing. Mice were killed by CO_2_ inhalation at different time points after wounding, and paraffin sections from the middle of the wounds were used for histological/histomorphometric analysis.

### 2.12 Statistical analysis

Analysis of mouse skin sections was performed blinded by the investigators. Statistical analysis was performed with PRISM software v5 (GraphPad Software Inc). Mann–Whitney *U* test was used for comparison between two different groups. *P > .05, **P > .01, *P > .001.

## 3 RESULTS

### 3.1 FGF receptors 1, 2 and 3 are expressed in the epidermis and in cultured keratinocytes

To determine whether the loss of FGFR1 and FGFR2 in keratinocytes can be partially compensated by other FGF receptors, we first analysed published data for *FGFR1, FGFR2, FGFR3 and FGFR4* expression levels. RNA sequencing data of FACS‐isolated cells from mouse skin at embryonic day 14.5 (E14.5) or postnatal day 5 (P5)^19^ revealed low expression levels of *FGFR1* in the epidermis, whereas *FGFR3* is expressed at intermediate levels and *FGFR2* at high levels. *FGFR4* expression was not detectable in the embryonic or neonate epidermis (Figure S1A,B). In addition, various FGF ligands are expressed in embryonic and neonate mouse skin, in particular *FGF2*, *FGF7*, *FGF10* and *FGF18*.^19^ These data are consistent with single‐cell RNA sequencing data of cells from adult mouse epidermis.^20^ The relatively high expression of *FGFR3* in the epidermis suggests that this type of receptor may function alone or redundantly with FGFR1 and FGFR2 in keratinocytes.

### 3.2 Loss of FGFR3 in keratinocytes does not affect epidermal homeostasis and wound healing

To determine the functional relevance of FGFR3 in keratinocytes, we generated mice lacking FGFR3 in this cell type (designated K5‐R3 mice). Mice with floxed *FGFR3* alleles^15^ were mated with transgenic mice expressing Cre recombinase in keratinocytes under control of the keratin 5 (K5) promoter^16^ to generate K5‐Cre, *Fgfr3^f/f^* mice (designated K5‐R3 mice). K5‐R3 mice were viable and fertile and did not exhibit macroscopically visible abnormalities (Figure S2A). Histological analysis of the skin did not reveal obvious differences between K5‐R3 and Ctrl mice, with the exception of a slight increase in dermal thickness in some older FGFR3‐deficient mice (Figure S2B). There was no significant difference in the dermal thickness in 12‐week‐old mice (Figure S2C), and the density of the dermal matrix and the thickness of the adipose tissue were similar in mice of both genotypes at all ages (Figure S2B). As FGFR3 was specifically deleted in keratinocytes, we quantified the epidermal thickness and the number of proliferating keratinocytes in the epidermis (Ki67‐positive cells), but did not find a significant difference between genotypes (Figure S2D,E). Remarkably, even the healing of full‐thickness excisional wounds proceeded normally and there was no significant difference in wound closure or wound diameter (Figure S2F).

### 3.3 Generation of mice lacking all FGF receptors in keratinocytes

We next determined whether loss of FGFR3 aggravates the phenotype of mice lacking FGFR1 and FGFR2 in keratinocytes by generation of triple conditional knockout mice (K5‐R1/R2/R3 mice; see breeding scheme in Figure 1A). K5‐R1/R2/R3 mice were born with the expected Mendelian ratio. qRT‐PCR analysis of RNA from isolated epidermis of K5‐R1/R2/R3 mice confirmed the expression of Cre recombinase in the mutant mice and the efficient deletion of all three receptors (Figure 1B). The Cre‐mediated deletion of the *Fgfr3* floxed alleles results in loss of the IIIc exon and the exon encoding the transmembrane domain,^15^ and we confirmed the efficient deletion using primers that hybridize to this part of the mRNA. The major FGFR3 splice variant in the epidermis, however, is FGFR3b, which differs from FGFR3c in the second half of the third immunoglobulin (Ig)‐like domain and thus has different ligand‐binding specificities.^21^ The *FGFR3* knockout mice may still express a truncated RNA including the exon IIIb coding sequences, which, however, cannot give rise to a functional receptor because of the lack of the transmembrane domain. However, we also observed an efficient down‐regulation of FGFR3 mRNA in K5‐R3 and K5‐R1/R2/R3 mice using a primer hybridizing to exons 2/3, which should be present in such a truncated RNA. Therefore, such transcripts are most likely unstable. The strong reduction in FGFR3 mRNA levels also suggests that keratinocytes are the predominant producers of this receptor in the epidermis and that the expression in epidermal immune cells is negligible.

Efficient deletion of all FGF receptors was confirmed with cultured primary keratinocytes from the different mutant mice (Figure 1C). The loss of *FGFR1*, *FGFR2* and *FGFR3* did not result in a compensatory up‐regulation of *FGFR4*, and the mRNA levels of this receptor remained below the detection limit in mice of all genotypes. There was no compensatory up‐regulation of *FGFR3* in K5‐R1/R2 mice, but rather a reduced expression of this receptor (Figure 1B). Surprisingly, expression levels of *FGFR3* were highly variable in keratinocytes with wild‐type *FGFR3* alleles (Figure 1B) but this did not correlate with obvious phenotypic differences.

The complete loss of FGFR signalling in cultured keratinocytes from K5‐R1/R2/R3 mice was confirmed by proliferation studies with FGF1, which activates all FGFR variants.^22^ Whereas cells from control mice incorporated BrdU in response to FGF1 and to epidermal growth factor (EGF)—used here as positive control—keratinocytes from K5‐R1/R2/R3 mice only responded to EGF (Figure 1D).

### 3.4 Loss of FGFR3 in keratinocytes does not aggravate the macroscopic and histological phenotype of K5‐R1/R2 mice

**Figure 2:**
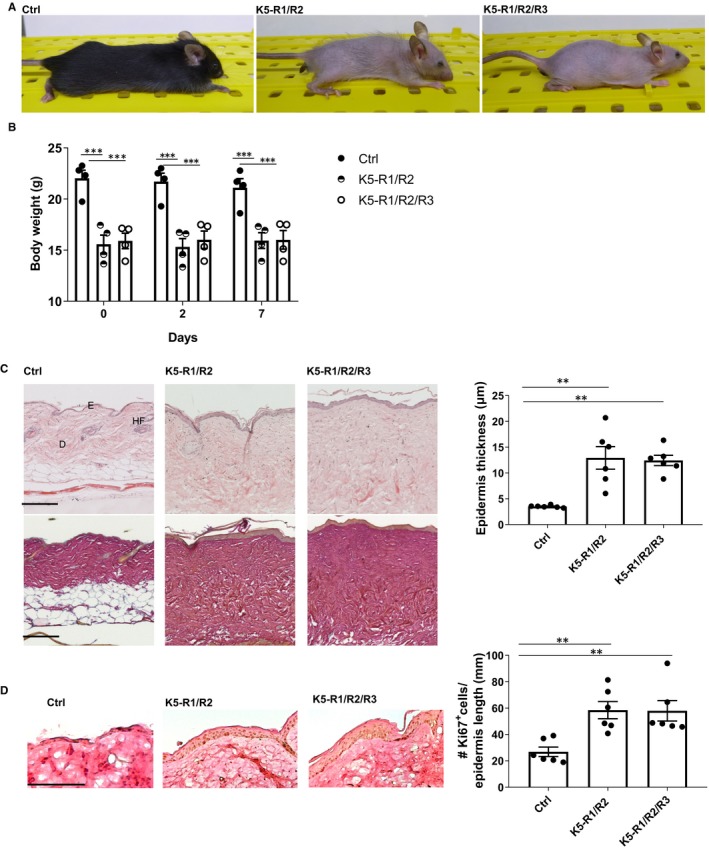
Loss of FGFR3 in keratinocytes does not aggravate the macroscopic phenotype of K5‐R1/R2 mice. A, Representative photographs of K5‐R1/R2/R3 mice and their control littermates lacking Cre recombinase (R1/R2 mice). B, Bodyweight of Ctrl, K5‐R1/R2 and K5‐R1/R2/R3 mice at the age of 12 weeks. N = 4 per genotype. C, Representative photomicrographs of haematoxylin/eosin (upper panel)‐ and Herovici‐stained (lower panel) paraffin sections from back skin of 9‐month‐old mice and their control littermates (left panel) and quantification of epidermal thickness (right panel). Magnification bars: 100 μm. D: dermis; E: epidermis, HF: hair follicles. D, Representative images of Ki67‐stained paraffin sections from back skin of 9‐month‐old mice and their control littermates (left panel) and quantification of Ki67‐positive cells per length epidermis (right panel). Magnification bars: 100 μm. D: dermis; E: epidermis, HF: hair follicles. Bars indicate mean ± SE in all graphs. N = 4‐6 per genotype. **P ≤ .01, ***P ≤ .001. Mann–Whitney *U* test

K5‐R1/R2/R3 mice were macroscopically similar to K5‐R1/R2 mice, including the progressive hair loss and the small body size (Figure 2A). Furthermore, K5‐R1/R2 and K5‐R1/R2/R3 mice had a similar reduction in bodyweight compared with control mice (Figure 2B).

Histological analysis of the skin showed the characteristic phenotype of K5‐R1/R2 mice with epidermal thickening.^4^ Again, this was not obviously aggravated by the additional loss of FGFR3 (Figure 2C). In addition, keratinocyte proliferation and differentiation as revealed by Ki67 immunohistochemistry (Figure 2D) or immunofluorescence analyses for differentiation‐specific proteins (Figure S3) were similar in K5‐R1/R2/R3 and K5‐R1/R2 mice. As previously shown,^4^ K5‐R1/R2 mice exhibited interfollicular expression of keratin 6 (K6), a sign for abnormal keratinocyte differentiation and enhanced proliferation.^23^ This was also not further aggravated by the loss of FGFR3 and also not detected in mice lacking FGFR3 alone, and the other differentiation‐specific keratins as well as the late differentiation marker loricrin were normally expressed in K5‐R3, K5‐R1/R2 and K5‐R1/R2/R3 mice (Figure S3).

### 3.5 Loss of FGFR3 further aggravates the epidermal barrier deficiency of K5‐R1/R2 mice

**Figure 3:**
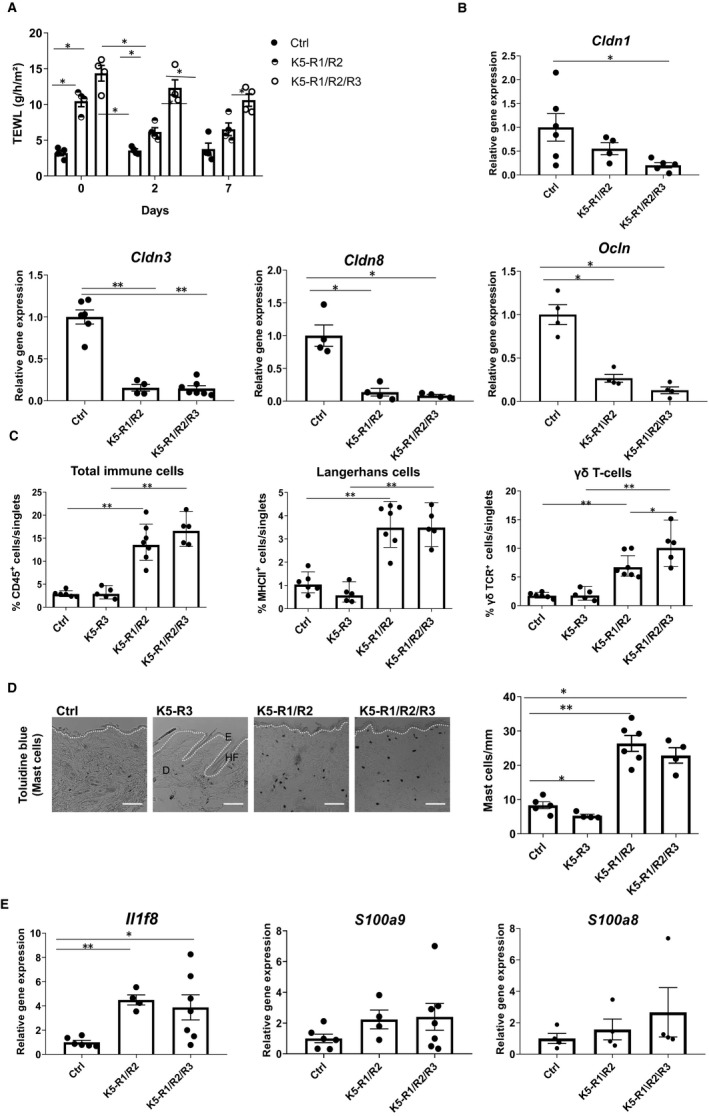
Loss of FGFR3 in keratinocytes mildly aggravates the barrier function defect of K5‐R1/R2 mice. A, Transepidermal water loss (TEWL) on the back skin of 9‐week‐old Ctrl, K5‐R1/R2 and K5‐R1/R2/R3 mice (in g/h/m^2^). Measurements were repeated on the same mice 2 and 7 days later. N = 4 mice per genotype. B, qRT‐PCR analysis of RNA samples from the epidermis of Ctrl, K5‐R1/R2 and K5‐R1/R2/R3 mice for *Cldn1* and *Cldn3* relative to *Rps29*. N = 4‐6 per genotype. C, Flow cytometry analysis of dissociated epidermal cells from Ctrl, K5‐R3, K5‐R1/R2 and K5‐R1/R2/R3 mice for quantification of total CD45^+^ immune cells, MHCII^+^ Langerhans cells and γδ T cells (DETCs). N = 5‐7 per genotype. D, Representative images of toluidine blue–stained paraffin sections from back skin and quantification of toluidine blue–stained mast cells/ mm dermis. The dotted white line indicates the basement membrane. Magnification bars: 50 μm. D: dermis, E: epidermis, HF: hair follicle. E, qRT‐PCR analysis of RNA samples from the epidermis for *Il1f8, S100a8* and *S100a9* relative to *Rps29*. N = 4‐6 per genotype. Bars indicate mean ± SE in all graphs. *P ≤ .05, **P ≤ .01. Mann–Whitney *U* test

A hallmark of the phenotype of K5‐R1/R2 mice is the defect in epidermal barrier function, which results at least in part from reduced expression of tight junction proteins and which causes a chronic inflammatory skin disease.^4^, ^6^, ^7^, ^8^ The transepidermal water loss (TEWL), which reflects the integrity of the inside‐out barrier, was indeed significantly higher in K5‐R1/R2/R3 compared with K5‐R1/R2 mice (Figure 3A). This was associated with a mild, but non‐significant reduction in the mRNA levels of the tight junction proteins claudin 1 (*Cldn1*) and occludin (*Ocln*), whereas *Cldn3* and *Cldn8* were expressed at equally low levels in mice of both genotypes (Figure 3B).

Flow cytometry analysis demonstrated that the number of total immune cells, Langerhans cells (MHCII‐positive cells in the epidermis) and epidermal γδ T cells (dendritic epidermal T cells (DETC)) was equally low in control *vs*. K5‐R3 mice (Figure 3C, and Figure S4 for flow cytometry), and the number of toluidine blue–positive mast cells was even mildly reduced by the loss of FGFR3 (Figure 3D). These immune cells were analysed, because their numbers are strongly increased in K5‐R1/R2 vs control mice^4^, ^6^, ^7^ (Figure 3C). With the exception of a significant increase in epidermal γδ T cells in K5‐R1/R2/R3 vs K5‐R1/R2 mice, the additional loss of FGFR3 did not further affect the number of the other immune cell types (Figure 3C). Consistent with the similar number of immune cells in K5‐R1/R2 and K5‐R1/R2/R3 mice, the genes encoding the pro‐inflammatory cytokines IL‐36β (=IL1‐F8) (*Il1f8*), S100A8 (*S100a8*) and S100A9 (*S100a9*) were expressed at similar levels in mice of both genotypes (Figure 3E).

### 3.6 FGFR deficiency in the epidermis causes dermal fibrosis

**Figure 4:**
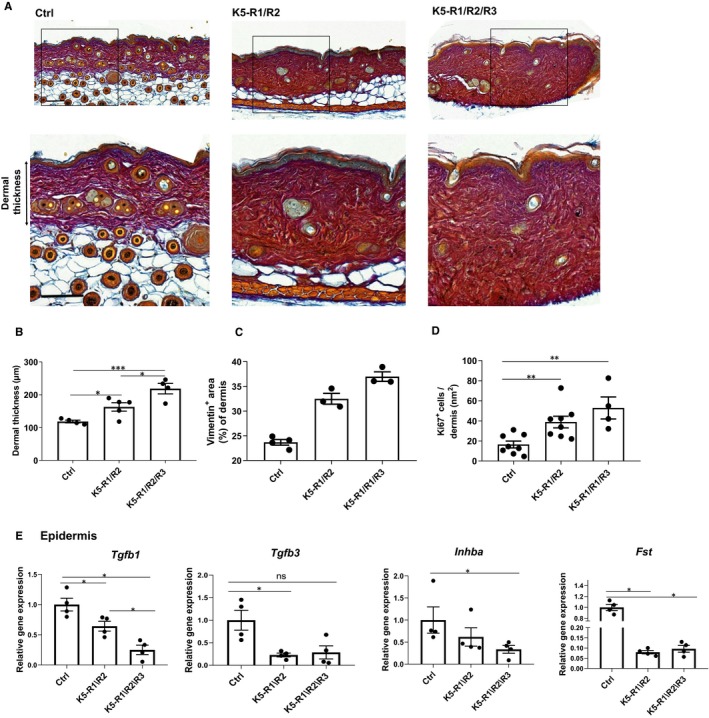
Loss of FGFR signalling in keratinocytes causes dermal fibrosis. A, Representative images of Herovici‐stained sections of 3‐month‐old Ctrl, K5‐R1/R2 and K5‐R1/R2/R3 mice. Magnification bars: 100 μm. Note the replacement of adipose tissue by fibrous tissue in the FGFR double and triple mutant mice. Squares indicate the area where the high magnification image was taken (lower panel). B, Quantification of dermal thickness in Ctrl, K5‐R1/R2 and K5‐R1/R2/R3 mice. N = 4 per genotype. C, Quantification of the percentage of vimentin‐positive skin area in Ctrl, K5‐R1/R2 and K5‐R1/R2/R3 mice. Epidermal area was excluded. N = 3‐4 per genotype. D, Quantification of Ki67‐positive cells in the dermis and subcutis of Ctrl, K5‐R1/R2 and K5‐R1/R2/R3 mice. N = 4‐8 per genotype. E, qRT‐PCR analysis of RNA samples from the epidermis of Ctrl, K5‐R1/R2 and K5‐R1/R2/R3 mice for *Tgfb1, Tgfb3, Inhba* and *Fst* relative to *Rps29*. N = 4 per genotype. Mean expression in Ctrl mice was set to 1. Bars indicate mean ± SE in all graphs. *P ≤ .05, **P ≤ .01, ***P ≤ .001. Mann–Whitney *U* test

An additional hallmark of the phenotype of K5‐R1/R2 mice, which we had previously not characterized, is the development of skin fibrosis. This is reflected by the enhanced dermal thickness and the presence of a dense connective tissue characterized by high levels of collagen, which had replaced the adipose tissue (Figure 4A). The fibrotic phenotype was particularly pronounced in K5‐R1/R2/R3 mice, and there was a further significant increase in dermal thickness compared with K5‐R1/R2 mice (Figure 4B). The percentage of dermal area that stained positive for vimentin was also higher in K5‐R1/R2/R3 compared with K5‐R1/R2 mice (Figure 4C), suggesting an increase in the number of mesenchymal cells in the dermis. Consistent with this finding, the number of dermal cells expressing the proliferation marker Ki67 was higher in K5‐R1/R2 compared with control mice and further increased in K5‐R1/R2/R3 mice (Figure 4D).

Overall, these findings suggest that the defect in the epidermal barrier affects the fibroblast phenotype, possibly through release of pro‐fibrotic factors. To test this possibility, we analysed the expression of *Tgfb1*, *Tgfb3*, *Inhba* and *Fst*, which give rise to the secreted factors TGF‐β1, TGF‐β3, activin A and the activin antagonist follistatin. Whereas expression of the pro‐fibrotic factors *Tgfb1, Tgfb3,* and *Inhba* was even moderately reduced in K5‐R1/R2 and K5‐R1/R2/R3 mice, *Fst* expression was strongly down‐regulated in mice of both genotypes, indicating a potential increase in biologically active activin (Figure 4E).

Taken together, these results demonstrate that loss of FGFR1/R2 in keratinocytes causes skin fibrosis, which is further aggravated upon loss of FGFR3.

## 4 DISCUSSION

Epidermal FGFR signalling plays a key role in skin homeostasis, repair and disease, but the contribution of the individual FGF receptors to different skin functions has remained largely unknown. Here, we show that FGFR2 is the major FGFR in the murine epidermis, whereas FGFR3 has only a minor supporting role. FGFR3 in keratinocytes is even dispensable for wound healing, in spite of its up‐regulation in wounded mouse skin.^24^ This is surprising, as activating mutations in this type of receptor cause acanthosis nigricans, seborrhoeic keratosis and epidermal naevi.^10^, ^11^, ^12^, ^13^ However, our findings are consistent with data from human keratinocytes demonstrating that knock‐down of FGFR3 does not affect normal keratinocyte growth in vitro,^25^ even though this receptor is strongly expressed in the human epidermis.^26^ These results point to a minor role of wild‐type FGFR3 in keratinocytes, at least under non‐challenged conditions.

Keratinocytes mainly express the IIIb splice variants of all FGF receptors, including FGFR3,^21^ and FGFR36 is rather poorly activated by most FGFs.^22^ By contrast, FGFR2b is strongly activated by FGF7 and FGF10, which are highly expressed in the skin.^19^, ^24^, ^27^ This provides a likely explanation for the particularly important role of FGFR2 vs FGFR1 and FGFR3 in keratinocytes. The unique role of FGFR2 is also reflected by the skin abnormalities seen in mice lacking only this receptor in keratinocytes,^4^, ^5^ which are aggravated, however, upon additional deletion of FGFR1.^4^

Our results further demonstrate that the loss of one FGF receptor in keratinocytes can be partially compensated by the other FGF receptors. Thus, FGFR1, FGFR2 and FGFR3 act together to maintain epidermal integrity, although the individual contributions are different. Finally, we show that loss of all FGF receptors in keratinocytes is compatible with life and with in vitro growth of keratinocytes. Therefore, other growth factor receptors are likely to compensate for the loss of FGFR signalling.

In spite of the keratinocyte‐specific deletion of FGF receptors, dermal fibrosis was seen in K5‐R1/R2 mice and was more severe in K5‐R1/R2/R3 mice. This may well be a consequence of the increase in immune cells that occurs in K5‐R1/R2 and also in K5‐R1/R2/R3 mice. However, additional loss of either mast cells or γδ T cells, two immune cell populations that are strongly increased in K5‐R1/R2 and in K5‐R1/R2/R3 mice, did not affect the dermal or epidermal phenotype in K5‐R1/R2 mice.^6^, ^7^ These findings suggest that fibroblasts are activated as a consequence of the epidermal abnormalities. Consistent with this assumption, it has been shown that a defect in the epidermal barrier results in up‐regulation of cytokines, such as S100A8, S100A9 and S100A12,^28^, ^29^, ^30^ which in turn cause fibroblast activation and fibrosis. S100A8 and S100A9 are also up‐regulated in K5‐R1/R2^4^, ^8^ and to a similar extent in K5‐R1/R2/R3 mice, and these cytokines likely contribute to the dermal fibrosis. Furthermore, we show here that expression of follistatin is strongly down‐regulated in the epidermis of K5‐R1/R2 and K5‐R1/R2/R3 vs control mice, which is likely to result in higher levels of bioactive activin, a potent pro‐fibrotic factor.^31^, ^32^ Therefore, the epidermal abnormalities seen in our FGFR mutant mice and also in patients with atopic dermatitis are likely to contribute to the development of skin fibrosis. Thus, amelioration of the epidermal alterations and the resulting inflammation is of crucial importance for the prevention of fibroblast activation, and activation of FGFR signalling in keratinocytes may be a promising strategy to improve the epidermal barrier and even to prevent/ameliorate the resulting dermal fibrosis.

## CONFLICT OF INTEREST

The authors confirm that there are no conflicts of interest.

## AUTHOR CONTRIBUTIONS

MM, MBG, TR, CS, MJ, HI and FK performed experiments and analysed the data. LC provided floxed FGFR3 mice. DO provided floxed FGFR1/FGFR2/FGFR3 mice. SW designed the study together with MM, wrote the manuscript and provided the funding. All authors made important suggestions to the manuscript.

## Data Availability

The data obtained in this study are shown in the figures and supplementary figures. Original data are available from the corresponding author upon reasonable request.
