# Supplementary figures and images for: Mouse genetics identifies unique and overlapping functions of fibroblast growth factor receptors in keratinocytes
Source: J Cell Mol Med. 2019 Dec 12;24(2):1774–85. doi: 10.1111/jcmm.14871 (PMC6991627; doi:10.1111/jcmm.14871)

Supplementary Figure S1

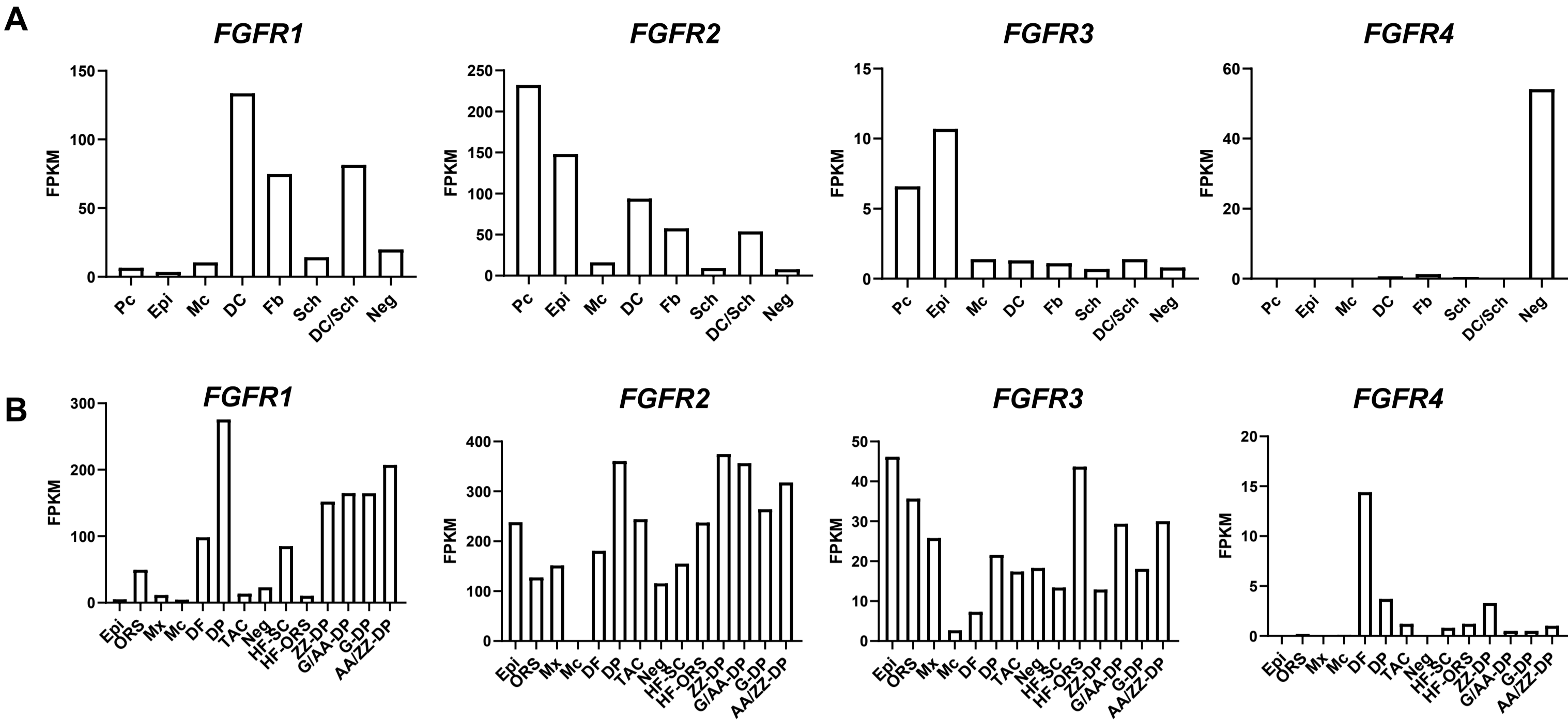

Supplement: Supplementary file 1 [file JCMM-24-1774-s001.pdf]

Supplementary Figure S2

A

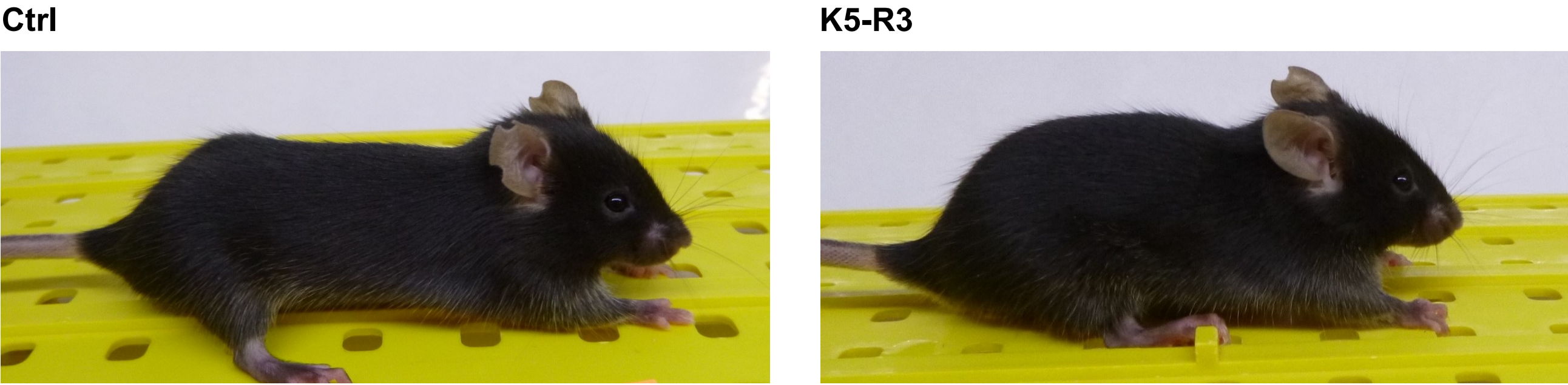

B

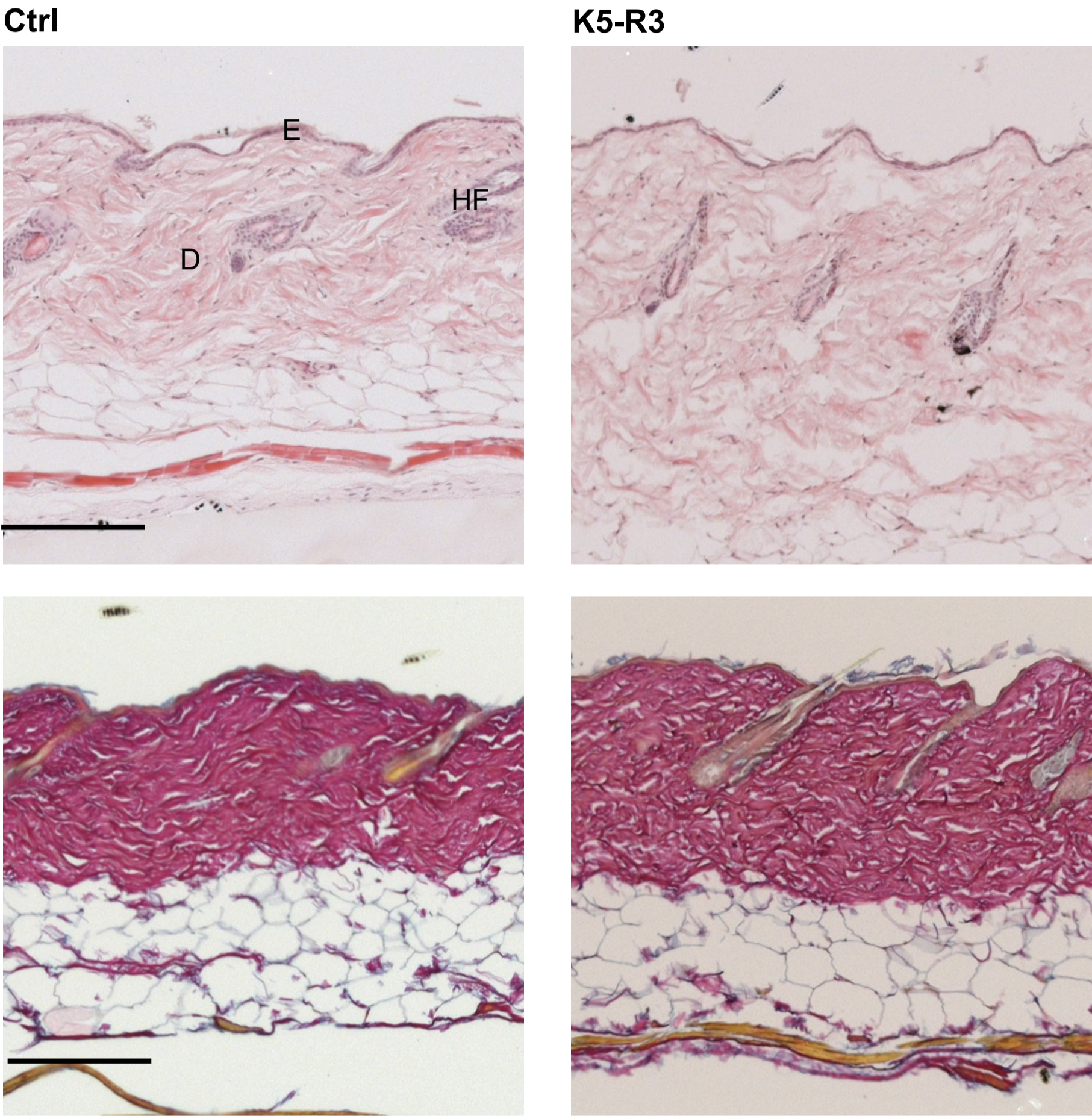

C

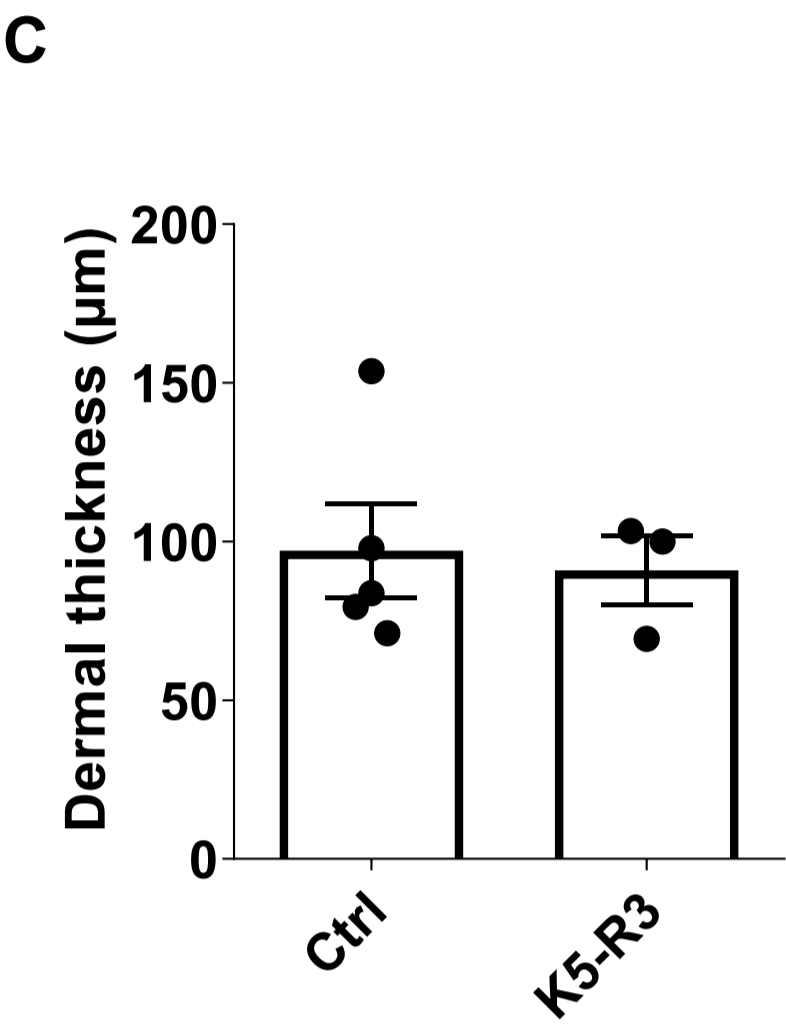

D

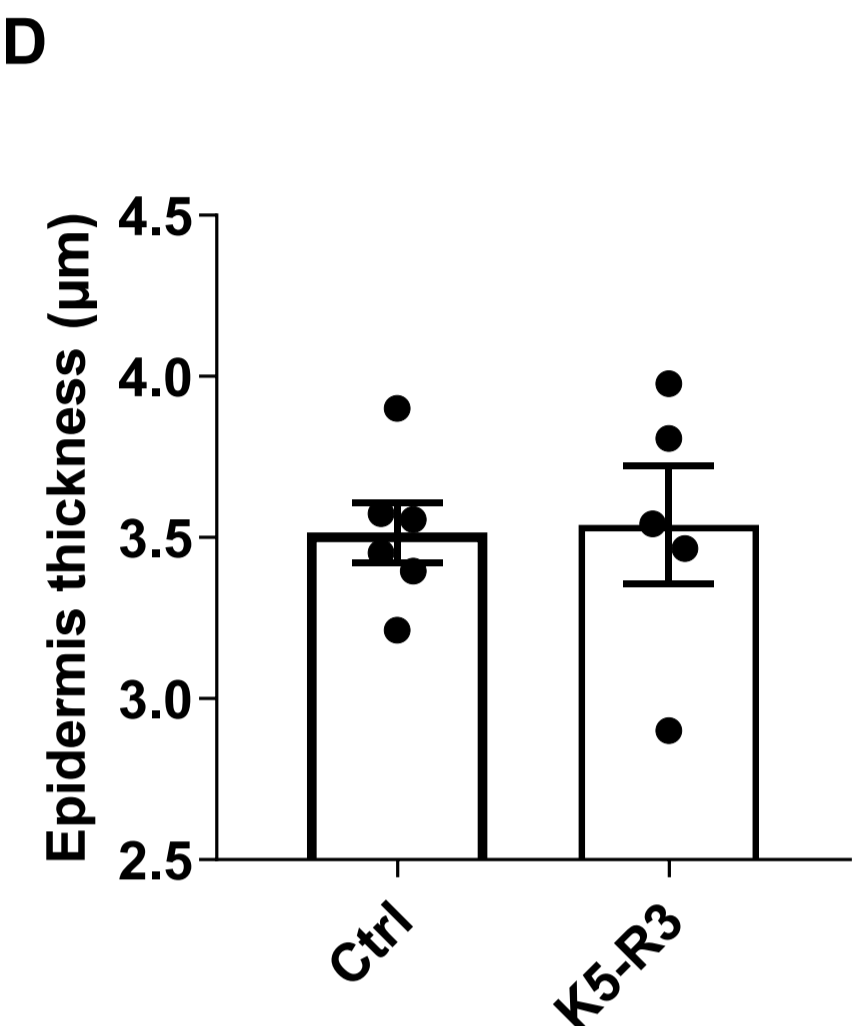

E

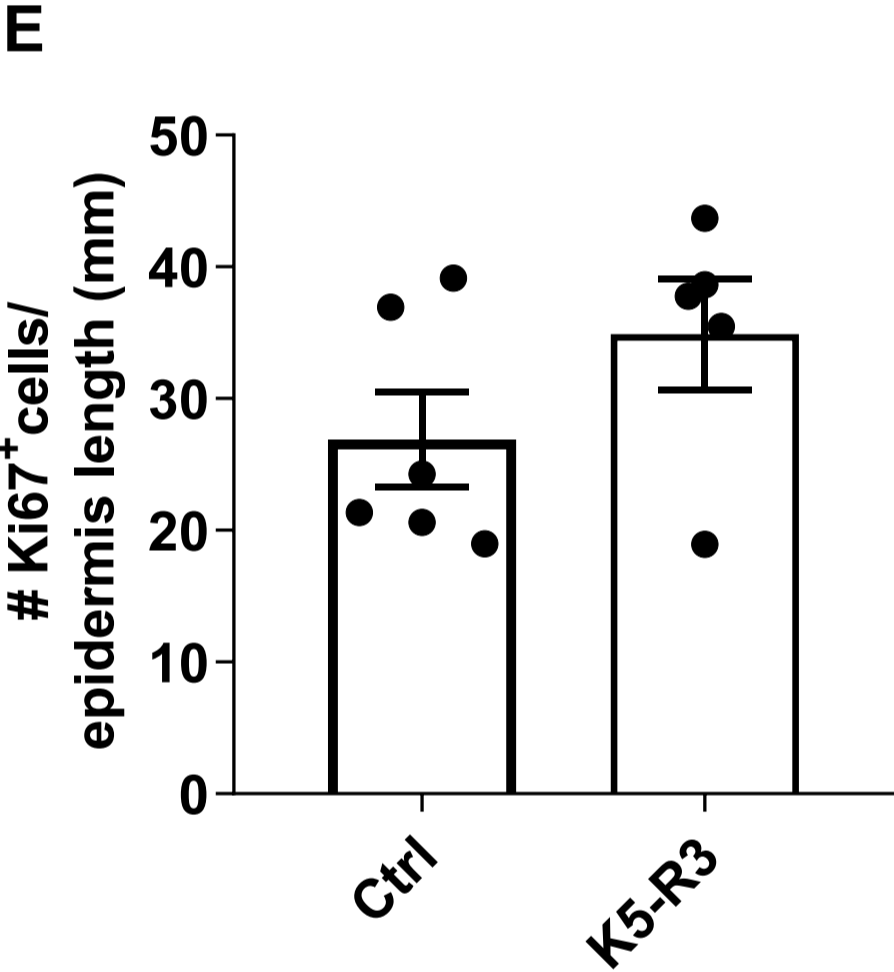

F

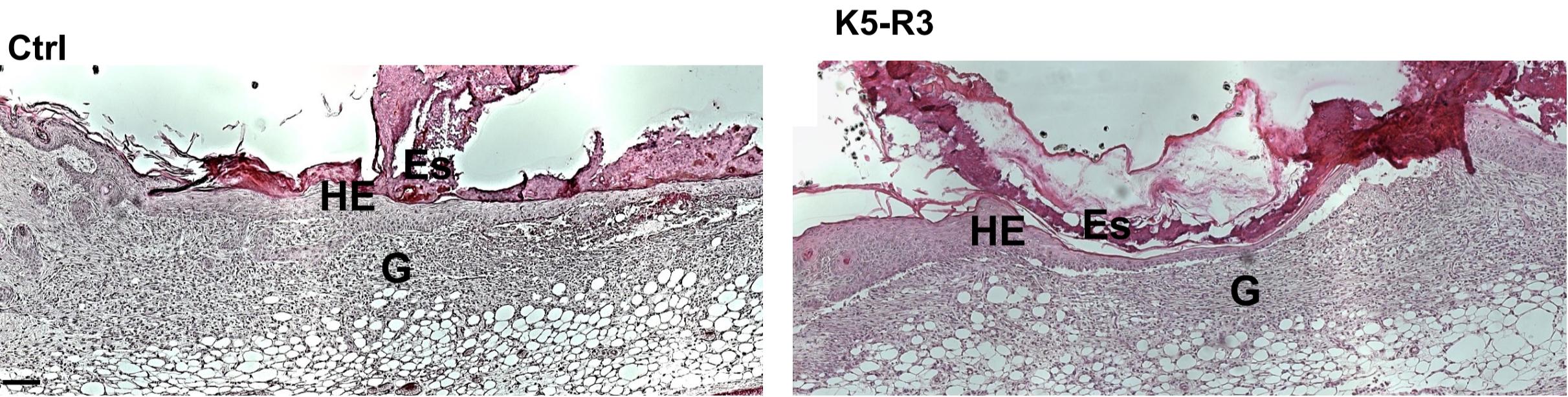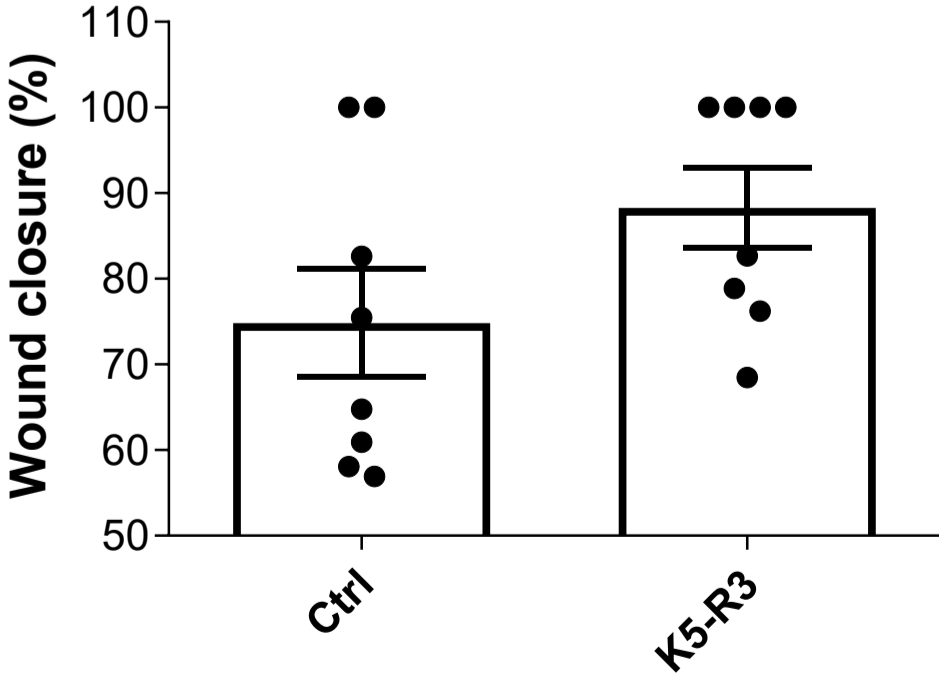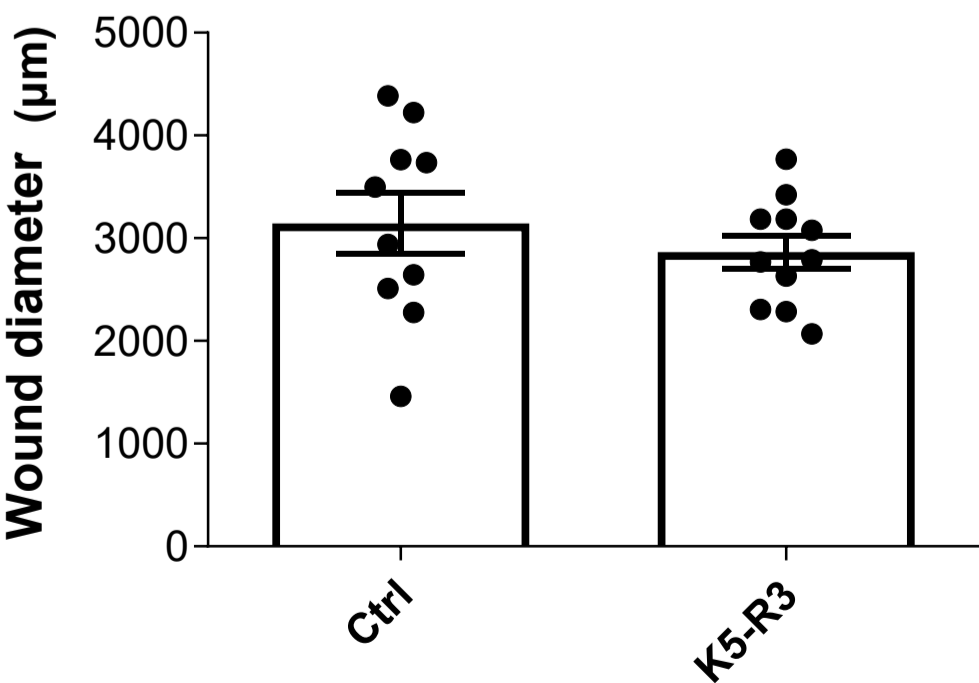

Supplement: Supplementary file 2 [file JCMM-24-1774-s002.pdf]

Supplementary Figure S3

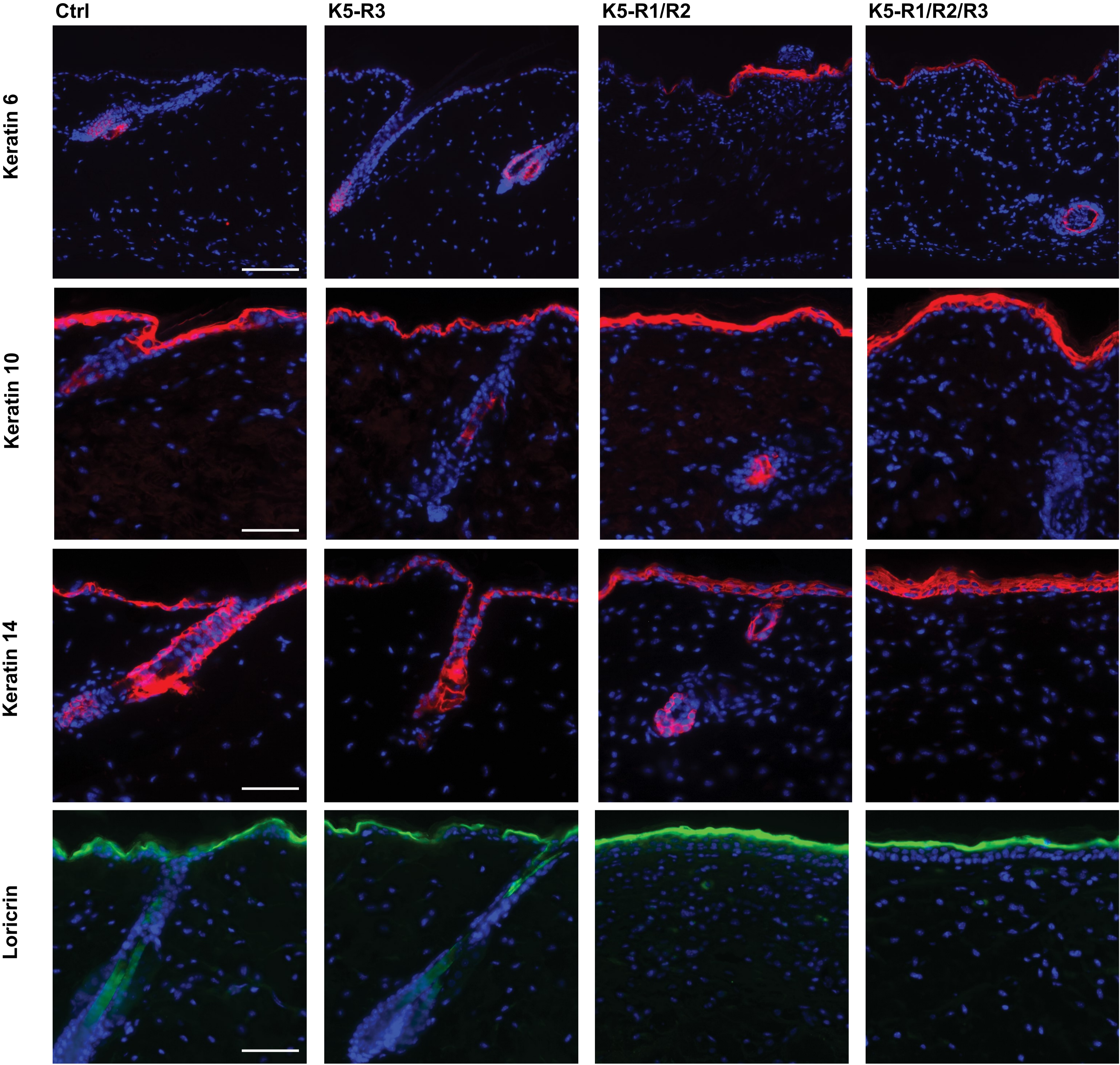

Supplement: Supplementary file 3 [file JCMM-24-1774-s003.pdf]

Supplementary Figure S4

A

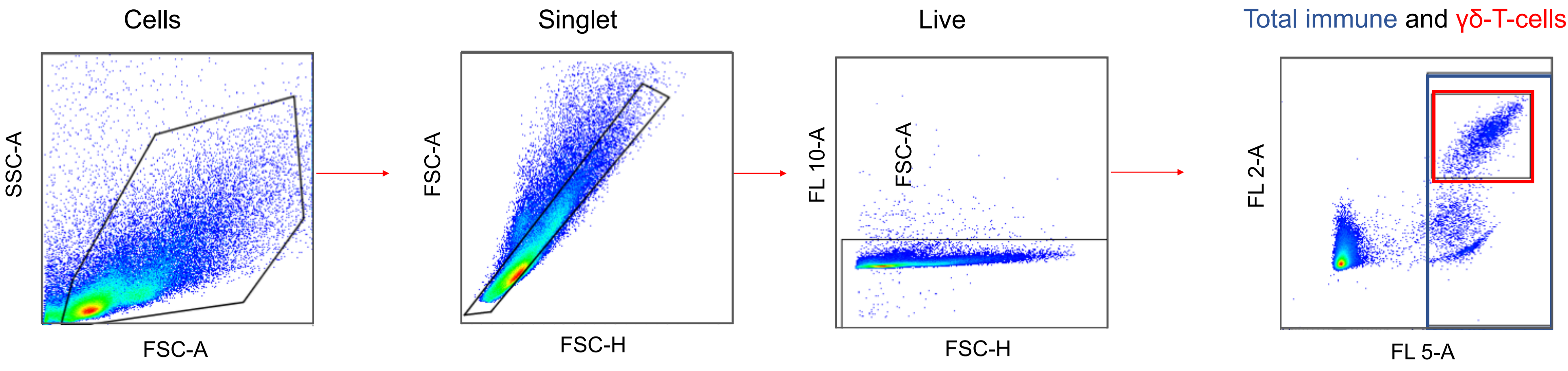

B

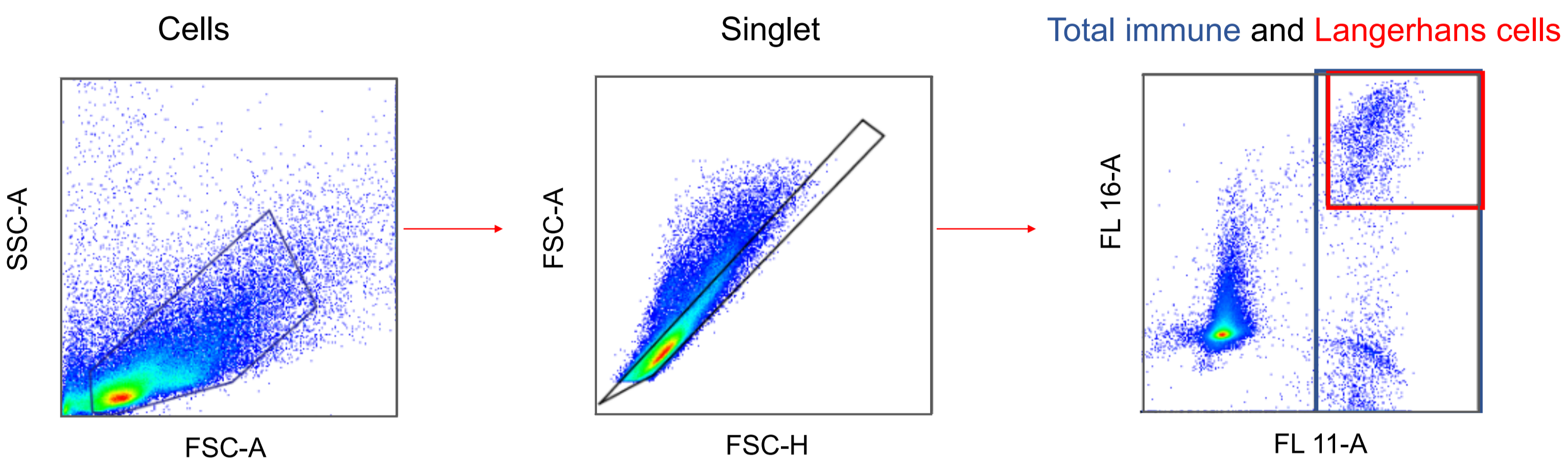

Supplement: Supplementary file 4 [file JCMM-24-1774-s004.pdf]
